# Severe Treatment-Refractory Macrophage Activation Syndrome: Hemophagocytic Lymphohistiocytosis Presenting With Neurologic Crisis in Systemic Lupus Erythematosus

**DOI:** 10.7759/cureus.106386

**Published:** 2026-04-03

**Authors:** Zehra Rahman, Tiffany Scotto, Shelby Watford, Brianna Woodbeck, Kabeer Ali, Parth Desai, Maleeha Abedi, Pramod Reddy

**Affiliations:** 1 Internal Medicine, University of Florida College of Medicine – Jacksonville, Jacksonville, USA; 2 Neurology, University of Florida College of Medicine – Jacksonville, Jacksonville, USA

**Keywords:** febrile status epilepticus, hemophagocytic lymphohistiocytosis (hlh), hyperferritinemia, lupus cerebritis, macrophage activating syndrome (mas), secondary hemophagocytic lymphohistiocytosis (hlh), systemic lupus erythromatosus

## Abstract

Hemophagocytic lymphohistiocytosis (HLH) is a life-threatening hyperinflammatory syndrome caused by uncontrolled activation of macrophages and cytotoxic lymphocytes, resulting in multi-organ dysfunction and high mortality if not promptly recognized. HLH can arise from genetic immune dysregulation or secondary to autoimmune disease, infection, or malignancy. When associated with autoimmune disease, it is referred to as macrophage activation syndrome (MAS).

We describe a 21-year-old woman with severe, complicated systemic lupus erythematosus (SLE) who presented with a month of progressive lethargy and episodic confusion that culminated in new-onset status epilepticus and severe neurologic involvement at admission. Laboratory studies and bone marrow biopsy confirmed MAS-HLH. The trigger was thought to be multifactorial, possibly related to recent initiation of cyclophosphamide, underlying severe autoimmune disease, and a preceding gastrointestinal illness.

Treatment of MAS-HLH with neurologic involvement is not standardized dure to the rarity of the disease. Current approaches include high-dose intravenous glucocorticoids, anakinra, intravenous immunoglobulin (IVIG), and plasma exchange, with variable responses. For refractory disease, the HLH-94 and HLH-2004 protocols, which are based on etoposide and dexamethasone, with or without cyclosporine, have been used. Our patient was initially managed with high-dose steroids and five days of plasma exchange, with inadequate clinical response. She was trialed on anakinra but ultimately required escalation to HLH-94/2004 protocol for disease control.

This case underscores the importance of maintaining high suspicion for MAS-HLH in patients with autoimmune disease and acute neurologic decline. Early recognition and timely escalation of therapy are critical to improving outcomes. Our patient highlights both the diagnostic challenges and therapeutic considerations in managing a rare and refractory case of MAS-HLH.

## Introduction

Hemophagocytic lymphohistiocytosis (HLH) is a rare, life-threatening hyperinflammatory syndrome characterized by uncontrolled immune activation and cytokine release that can progress rapidly without prompt recognition and treatment. HLH is broadly classified as either primary (genetic) or secondary to underlying conditions such as autoimmune disease, malignancy, or infection. When HLH occurs in association with autoimmune disease, it is referred to as macrophage activation syndrome (MAS), most commonly described in systemic lupus erythematosus (SLE), systemic juvenile idiopathic arthritis, adult-onset Still disease, and Kawasaki disease [1]. MAS-HLH typically presents with cytopenias, organ dysfunction, coagulopathy, and systemic inflammation driven by excessive macrophage activation.

Diagnosis remains challenging due to overlapping clinical features with sepsis and disease flares of the underlying autoimmune condition. Established diagnostic frameworks, including the HLH-2004 criteria and HScore, incorporate clinical findings and laboratory abnormalities such as fever, cytopenias, hyperferritinemia, hypertriglyceridemia, and hemophagocytosis, though these criteria were primarily developed in pediatric populations and may be less specific in adults [2]. Additionally, optimal treatment strategies for MAS-HLH remain incompletely standardized, particularly in refractory disease.

Here, we present a case of refractory MAS-HLH in a patient with SLE complicated by severe neurologic crisis, occurring in the absence of classic features of lupus flare. This case highlights diagnostic challenges in differentiating MAS-HLH from autoimmune disease activity and illustrates therapeutic considerations in treatment-resistant disease.

## Case presentation

A 21-year-old woman with a four-year history of SLE, complicated by lupus cerebritis, lupus nephritis, Raynaud’s phenomenon, and polyarthritis, presented with new-onset status epilepticus in the setting of two months of progressive encephalopathy. Her encephalopathy was characterized during this time by lethargy, worsening confusion or “brain fog,” headaches, and vision loss.

One month prior to admission, she was hospitalized at an outside facility for progressive confusion. Her laboratory analysis at that time revealed elevated lactate dehydrogenase (LDH, 602 IU/L; normal range: 126-266 IU/L), hyperferritinemia (1601 ng/mL; normal range: 15.0-150.0 ng/mL). Anti-nuclear antibody (ANA) was positive with a 1:1290 titer and a speckled pattern. She was treated with a three-day course of high-dose intravenous methylprednisolone (1000 mg daily) for presumed lupus cerebritis with minimal improvement and was discharged on oral prednisone 30 mg daily. After discharge, her maintenance hydroxychloroquine and mycophenolate were discontinued, and she was induced with intravenous cyclophosphamide [3], receiving the first infusion one week before the current admission. Additionally, she was also hospitalized one week prior to the current admission at an outside facility for severe enterocolitis, which resolved with intravenous fluids and broad-spectrum antibiotics.

On presentation for the current admission, the patient was severely encephalopathic and most of the history was obtained from her family. Her mother reported five tonic-clonic seizures on the morning of admission, three of which were witnessed by emergency medical personnel. On physical examination, the patient was alert but markedly encephalopathic, with slowed and delayed responses. She was oriented to person and time but not place. Cranial nerve testing showed impaired ocular tracking. Motor strength was 0/5 in all extremities. She followed simple commands but was unable to perform complex tasks.

**Figure 1:**
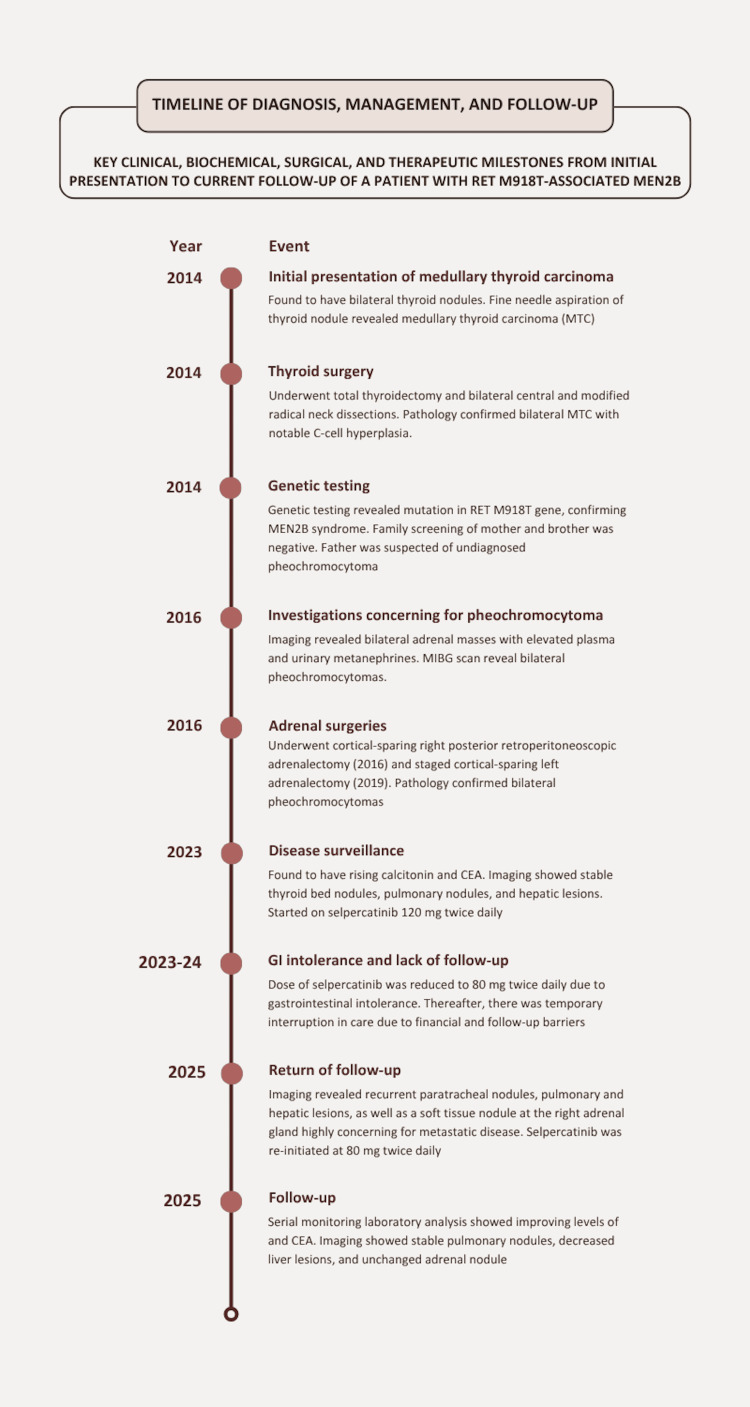
Timeline of SLE disease course and recent clinical events leading to the diagnosis of HLH SLE: systemic lupus erythematosus; HLH: hemophagocytic lymphohistiocytosis; MTC: medullary thyroid carcinoma; MIBG: metaiodobenzylguanidine: CEA: calcinoembryonic antigen. Image credit: Developed by authors using Canva (Canva Pty Ltd., Sydney, New South Wales, Australia).

A timeline summarizing her autoimmune disease progression over the past four years, along with recent clinical events leading to admission, is provided in Figure 1.

On admission, the patient was febrile (102.4°F or 39.1°C; normal range: 97°F (36.1°C) to 99°F (37.2°C)). Laboratory studies revealed lactic acidosis, transaminitis, and elevated lipase. Urinalysis showed hematuria. Complete blood count revealed pancytopenia with hemoglobin level of 8.5 g/dL (normal range: 12-16 g/dL), white blood cell count of 0.90 × 10³/µL (normal range: 4.5-11.0 ×10³/µL), and platelet count of 64 × 10³/µL (normal range: 140-440 x 10³/µL). Hemolysis workup demonstrated very low haptoglobin, a negative direct antiglobulin test (DAT), and no schistocytes on peripheral smear with an elevated fibrinogen level. A Disintegrin And Metalloproteinase with a ThromboSpondin type 1 motif, member 13 (ADAMTS13) activity was normal. LDH was markedly elevated from prior (1,060 IU/L; normal range: 126-266 IU/L). 

Inflammatory markers were notable for hyperferritinemia worse than prior (13,685 ng/mL; normal range: 15.0-150.0 ng/mL), hypertriglyceridemia (606 mg/dL, normal value: <=150 mg/dL), and elevated erythrocyte sedimentation rate (ESR) and C-reactive protein (CRP). Complement testing showed mildly decreased C3 with normal C4. ANA was positive with a titer of 1:640 and a speckled pattern. Anti-double stranded DNA was within normal limits. Objective laboratory analysis with reference ranges is presented in Table 1.

**Table 1:** Vital signs and pertinent laboratory analysis during hospital admission with HLH HLH: hemophagocytic lymphohistiocytosis; ESR: Erythrocyte sedimentation rate; CRP: C-reactive protein; CSF: Cerebrospinal fluid; ADAMTS13: A Disintegrin And Metalloproteinase with a ThromboSpondin type 1 motif, member 13.

Parameter	Result	Reference range
Blood pressure	148/118 mmHg	Less than 120/80 mmHg
Temperature (oral)	39.1 °C (102.4 °F)	97°F (36.1 °C) to 99°F (37.2°C)
Respiratory rate	21 bpm	12 to 20 breaths per minute
Oxygen saturation (SpO_2_)	99%	95% to 100%
Laboratory parameters	Result	Reference range
White Blood Cell Count (WBC)	0.90 ×10³/µL	4.5 – 11.0 ×10³/µL
Hemoglobin (Hb)	8.5 g/dL	12-16 g/dL
Platelet count	64 ×10³/µL	140-440 x ×10³/µL
Lactate dehydrogenase (LDH)	1060 IU/L	126-266 IU/L
Reticulocyte count	4.9%	0.5 - <1.5 %
Haptoglobin	<10 mg/dL	30 - 200 mg/dL
Fibrinogen	457 mg/dL	200 - 400 mg/dL
Direct antiglobulin test	Negative	Negative
ADAMSTS13 activity	83.5%	>66.8%
Lactic acid	6.3 mmol/L	0.7 - 2.0 mmol/L
Antinuclear antibody (ANA)	Positive, speckled pattern, titer 1:640	Negative
Anti-double stranded DNA (Anti-dsDNA)	5 IU/mL	<30 IU/mL
Ferritin	13,685 ng/mL	15.0 - 150.0 ng/mL
Triglyceride	606 mg/dL	≤150 mg/dL
Lipase	102 U/L	0 - 60 U/L
ESR	87 mm/hr	0-20 mm/hr
CRP	86.8 mg/L	0.10 - 5.00 mg/L
C3 complement	75 mg/dL	90 - 180 mg/dL
C4 complement	23 mg/dL	10 - 40 mg/dL
Aspartate aminotransferase (AST)	93 U/L	14 - 33 IU/L
Alanine aminotransferase (ALT)	46 U/L	10 - 42 IU/L
Soluble IL-2 Receptor (sCD25)	970 U/mL	158–623 U/mL
CXCL9	9,782 pg/mL	≤647 pg/mL
CSF analysis	Result	Reference range
Glucose	58 mg/dL	60 - 80 mg/dL
Protein	19 mg/dL	15 - 60 mg/dL
Gram stain and culture	Negative	Negative

**Figure 2:**
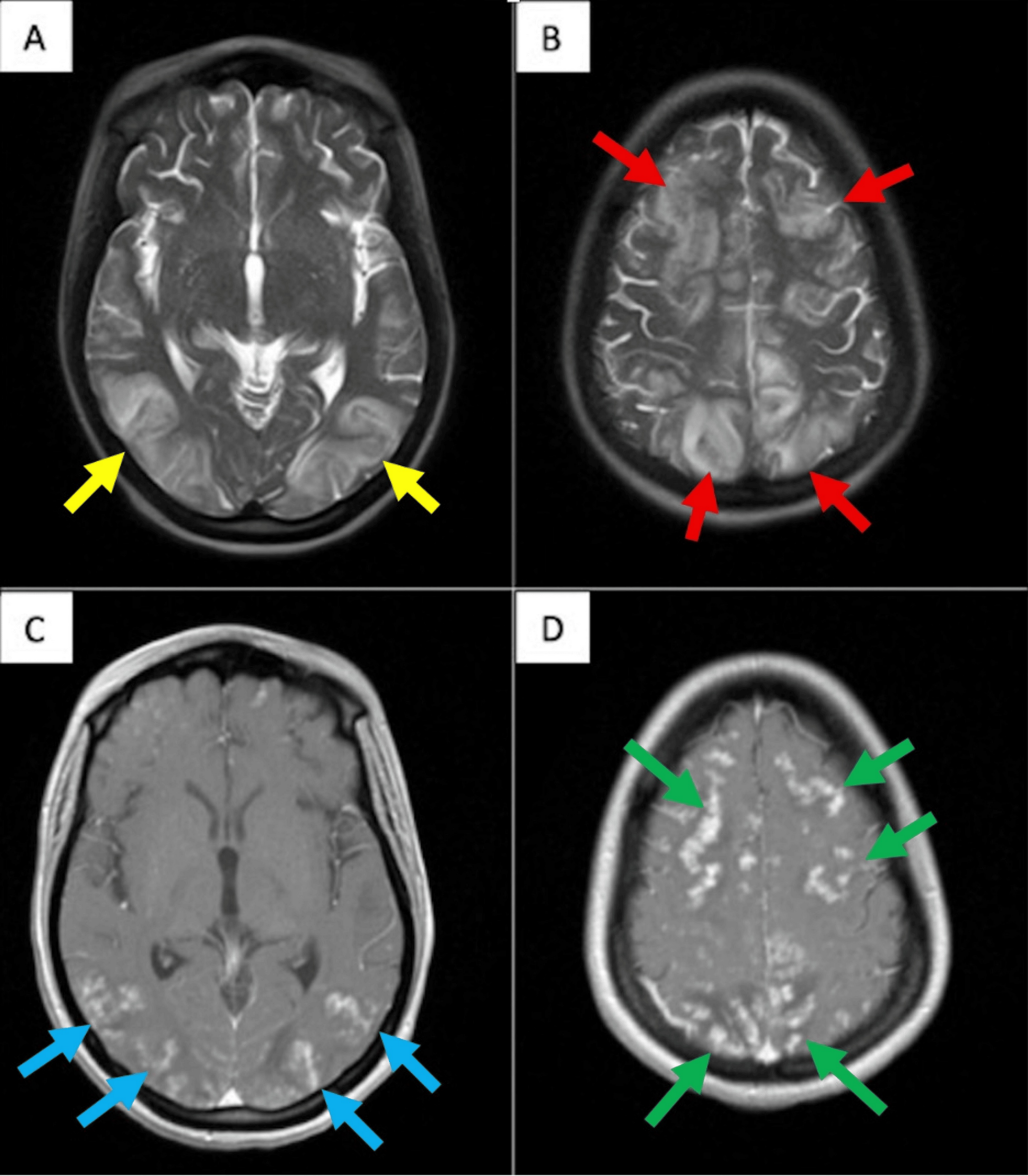
Brain MRI showing features atypical for posterior reversible encephalopathy syndrome (PRES) (A) Axial Half-Fourier Acquisition Single-Shot Turbo Spin-Echo (T2/HASTE) image showing bilateral parieto-occipital hyperintensities (yellow arrows); (B) Axial T2/HASTE image showing bilateral frontal and parietal hyperintensities (red arrows); (C) Post-contrast axial T1 image demonstrating nodular enhancement in the bilateral parieto-occipital lobes (blue arrows); (D) Post-contrast axial T1 image demonstrating nodular enhancement in the bilateral frontal and parietal lobes (green arrows).

Neurologic evaluation included a lumbar puncture, which was unrevealing: cerebrospinal fluid (CSF) glucose 58 mg/dL, protein 19 mg/dL, no nucleated cells, and negative Gram stain and culture. Brain MRI demonstrated bilateral cortical and subcortical hyperintensities on Half-Fourier Acquisition Single-Shot Turbo Spin-Echo (T2/HASTE) sequences (used in place of motion-degraded T2-weighted Fluid-Attenuated Inversion Recovery (T2/FLAIR)), predominantly involving the frontal and occipital lobes, which was initially concerning for posterior reversible encephalopathy syndrome (PRES). However, post-contrast T1 images revealed diffuse nodular enhancement in the frontal, parietal, and occipital lobes, which is atypical for PRES. These MRI findings are depicted in Figure 2.

Continuous electroencephalogram (EEG) showed an active epileptic focus in the right hemisphere with generalized slowing, consistent with severe diffuse encephalopathy of nonspecific etiology.

**Figure 3:**
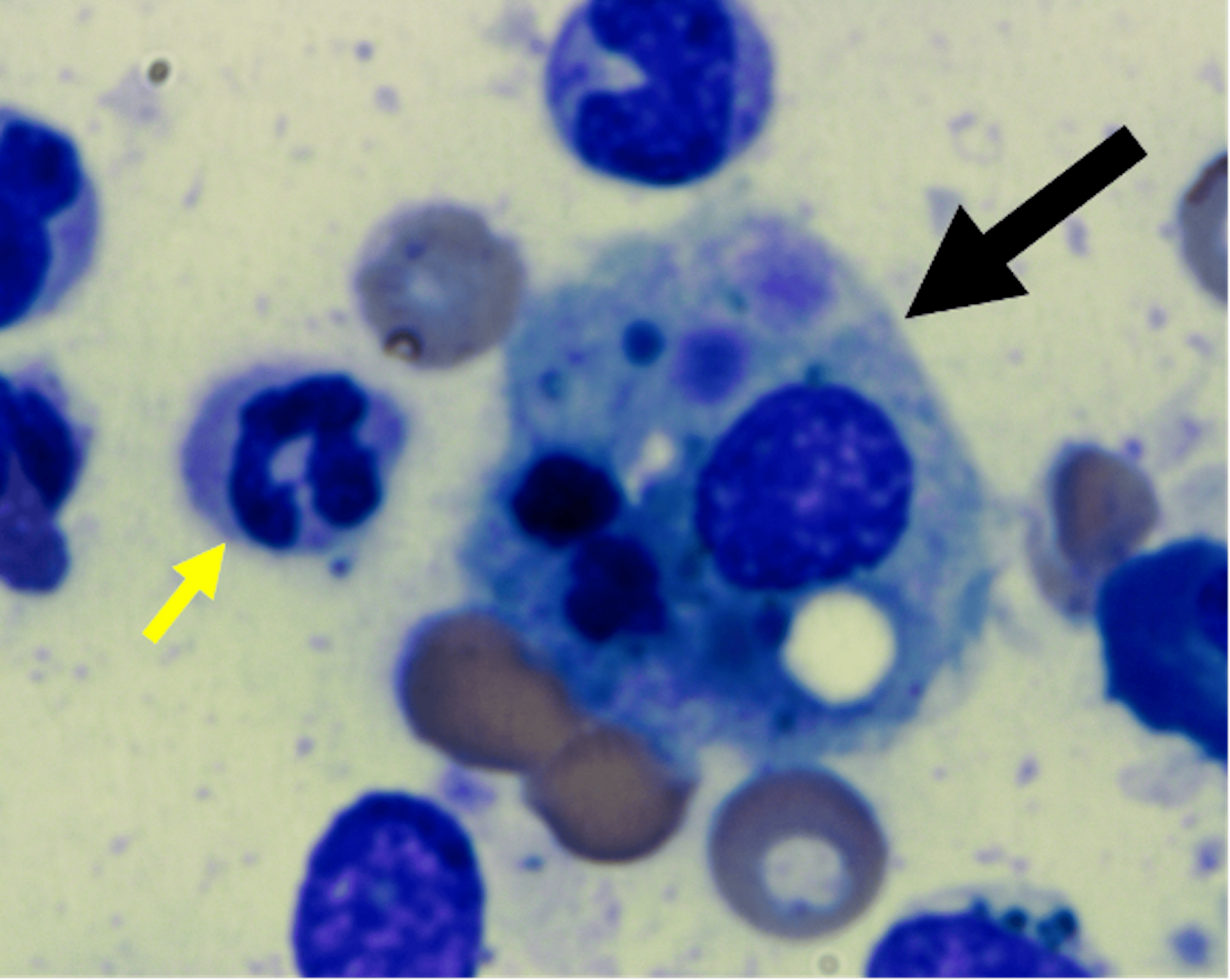
Bone marrow aspirate smear showing a hemophagocytic histiocyte with engulfed hematopoietic cells (large black arrow) The large macrophage demonstrates abundant cytoplasm containing erythroid precursors and leukocytes, consistent with hemophagocytosis. Surrounding cells include neutrophils (small yellow arrow) and other marrow elements. Wright–Giemsa stain, ×1000.

Given the marked hyperferritinemia, evaluation for HLH was pursued. Soluble IL-2 receptor (sCD25) was mildly elevated (970 U/mL), while CXCL9 was markedly elevated (9,782 pg/mL). Bone marrow biopsy demonstrated hemophagocytic histiocytes within a hypocellular marrow (50%) with trilineage hematopoiesis, without increased blasts or significant dysplasia (Figure 3). 

Although detailed laboratory values are presented in Table 1, synthesis of key findings demonstrated that this patient met multiple diagnostic criteria for HLH, including high-grade fever, cytopenias affecting all three lineages, marked hyperferritinemia (13,685 ng/mL), hypertriglyceridemia (606 mg/dL), transaminitis, and hemophagocytosis on bone marrow biopsy. Her calculated HScore was 246, corresponding to a >99% probability of HLH [2], well above the commonly used diagnostic threshold (≥169). These findings fulfill multiple components of the HLH-2004 diagnostic criteria, supporting the diagnosis of HLH in this context.

Differential diagnoses were carefully considered. A lupus flare was initially suspected given her underlying SLE, neurologic symptoms, and history of lupus cerebritis. Thrombotic microangiopathy (TMA) was also considered due to cytopenias, elevated LDH, and evidence of hemolysis; however, absence of schistocytes and normal ADAMTS13 activity argued against TMA.

Potential HLH triggers were explored. The patient had a recent episode of enterocolitis, which may have provided an infectious or inflammatory stimulus. Additionally, initiation of intravenous cyclophosphamide one week prior could have precipitated HLH, as immunosuppressive therapy has been reported to trigger hyperinflammatory syndromes in susceptible patients. Recognition of these possible triggers helped contextualize the rapid progression of her clinical course and supported prompt initiation of HLH-directed therapy.

Initial management included broad-spectrum antibiotics and high-dose intravenous methylprednisolone (1000 mg daily for five days). Lumbar puncture was unrevealing for infectious etiology, with normal cell counts, and antibiotics were subsequently discontinued. The patient was admitted to the neurointensive care unit for status epilepticus, intubated, and sedated with propofol. She received antiepileptic therapy, resulting in resolution of the previously observed epileptic focus on continuous EEG.

Given persistent cytopenias, hyperferritinemia, and worsening encephalopathy, plasma exchange was initiated and continued for five days. Anakinra, an interleukin-1 receptor antagonist, was subsequently added, leading to extubation and notable improvement in neurologic status. At that time, she was alert and oriented, though not at her baseline, with delayed responsiveness and lethargy. Despite partial neurologic recovery, disease-specific markers (LDH, ferritin) remained elevated, with suppressed haptoglobin and progressive pancytopenia.

Because she demonstrated inadequate clinical and laboratory response despite high-dose glucocorticoids, plasma exchange, and anakinra, therapy was escalated to the HLH-94 protocol with etoposide and dexamethasone. However, she experienced severe cytopenias, requiring intermittent pauses in therapy.

The patient’s clinical course is ongoing. There are plans for emapalumab if she remains refractory, with possible re-introduction of anakinra in combination with etoposide when tolerated. Once recovery from the acute HLH phase is achieved, she is planned to have longitudinal follow-up for consideration of non-malignant allogeneic hematopoietic stem cell transplant.

## Discussion

In this case, the diagnosis of MAS-HLH was strongly supported by both HLH-2004 criteria and HScore [2]. The patient fulfilled key criteria including fever, cytopenias, hyperferritinemia, hypertriglyceridemia, and hemophagocytosis on bone marrow biopsy. Her HScore of 246 corresponds to a greater than 99% probability of HLH, reinforcing diagnostic certainty despite overlapping features with lupus flare [2].

Importantly, distinguishing MAS-HLH from active SLE remained challenging, as both conditions share features such as cytopenias and systemic inflammation; however, the degree of hyperferritinemia and presence of hemophagocytosis favored HLH.

The North American Consortium for Histiocytosis reports that MAS occurs in approximately 4% of patients with rheumatic diseases such as SLE [4]. Initial management of MAS-HLH typically involves high-dose intravenous glucocorticoids, with monitoring of clinical and laboratory response before escalation. Adjunctive agents, including anakinra, IVIG, and corticosteroids, have been used with reported benefit [5]. Disease-specific markers such as ferritin, platelet count, liver enzymes, triglycerides, LDH, fibrinogen, and D-dimer are recommended for monitoring disease activity and therapeutic response [6].

In our patient, urgent treatment was initiated with high-dose intravenous methylprednisolone and plasma exchange for five days during admission to the neurointensive care unit. Anakinra was added given its favorable safety profile, rapid onset, and adequate central nervous system (CNS) penetration in adults [1]. Although randomized controlled trials are lacking, observational and open-label multicenter studies have demonstrated that anakinra can be an effective adjunctive therapy in MAS-HLH, providing rapid cytokine blockade with acceptable safety in critically ill patients [7,8]. Despite some clinical improvement with extubation, the patient’s neurologic recovery remained limited and disease markers continued to worsen.

Cyclosporine, a calcineurin inhibitor, was considered for combination therapy due to its efficacy in suppressing hyperinflammation in secondary MAS-HLH [9]. However, it was withheld due to potential nephrotoxicity, hypertension, increased infection risk, PRES, bone marrow suppression, and hepatotoxicity [1,10].

For patients with treatment-refractory or relapsed MAS-HLH, evidenced by worsening disease-specific markers or clinical deterioration such as fever, organomegaly, neurologic findings, hepatitis, or pancytopenia, management is typically escalated to the HLH-94 protocol, which includes etoposide (VP-16) and dexamethasone [1]. HLH-94 is considered the standard of care for primary HLH, with HLH-2004 building upon the initial study [1]. Etoposide selectively depletes activated T cells that drive the cytokine storm in HLH [11] and is reserved for severe, refractory disease. Although designed for primary HLH, these regimens have been extended to severe secondary MAS-HLH [12]. The protocols also allow for cyclosporine, with earlier introduction in HLH-2004; however, its toxicity profile can limit use, as discussed previously.

In this patient, a stepwise approach was employed: intravenous glucocorticoids, plasma exchange, and anakinra were initiated first, followed by HLH-94 when clinical and laboratory markers continued to worsen. Current consensus favors early and aggressive HLH protocol initiation in MAS-HLH with severe neurologic involvement to prevent irreversible damage. However, given the rarity of such severe cases, there is no clear consensus on whether upfront HLH protocol or a stepwise approach is superior, as early cytotoxic therapy carries significant risk. Prompt bone marrow confirmation in this patient allowed for rapid initiation of the nine-week HLH protocol once refractory disease was established.

For patients intolerant of conventional HLH regimens or with progressive disease, emapalumab, a monoclonal antibody targeting interferon-gamma, has emerged as a therapeutic option. In this case, etoposide was intermittently held due to severe myelosuppression, prompting consideration of emapalumab if the patient remains refractory. The pivotal NI-0501 trial evaluated emapalumab in 34 patients with primary HLH refractory to conventional therapy, demonstrating disease control sufficient to allow most to proceed to hematopoietic stem cell transplantation [13]. Additional reports support its use in relapsed or refractory HLH, stabilizing disease and facilitating transition to transplant [14]. For our patient, emapalumab remains under consideration as a bridging therapy; her clinical course is ongoing, and she has not yet achieved meaningful neurologic recovery despite aggressive management.

## Conclusions

Secondary MAS-HLH in the setting of SLE is a rare, life-threatening condition that requires prompt recognition and aggressive treatment to prevent mortality, particularly when central nervous system involvement is present, as delayed intervention can lead to irreversible neurologic damage. In this case, MAS-HLH was likely multifactorial, with suspected triggers including initiation of cyclophosphamide in the context of severe autoimmune disease and recent gastrointestinal illness.

First-line therapy with high-dose intravenous glucocorticoids, supplemented by adjuncts such as IVIG, anakinra, and plasmapheresis, remains the cornerstone of early management, while disease-specific markers must be closely monitored to guide treatment escalation. Although cyclosporine can effectively suppress hyperinflammation, its toxicity profile limits its use in critically ill patients. For refractory or relapsed disease, HLH-94/HLH-2004 protocols with etoposide and dexamethasone remain standard, but associated toxicities are significant and comparative studies of alternative regimens are lacking. Emerging agents such as emapalumab offer novel immunomodulatory options and may bridge patients to hematopoietic stem cell transplantation.

This case highlights the complexity of MAS-HLH management, the challenges of treatment-refractory disease, and evolving therapeutic strategies that continue to shape our understanding of this high-mortality syndrome.
